# GP perceptions of informal peer support in primary care: a qualitative study

**DOI:** 10.3399/BJGPO.2023.0151

**Published:** 2024-04-17

**Authors:** Aminah Hussan, Maya Satheeskaran, Hajin Dho, Avishek Basu, Yunis Fazaldin, Samir Tariq, Maria Farkas

**Affiliations:** 1 Imperial College Business School, Imperial College London, London, UK

**Keywords:** peer support, general practitioners, burnout, psychological, qualitative research

## Abstract

**Background:**

Burnout is on the rise among GPs in the UK. One approach to mitigating burnout in GPs is through informal peer support (IPS). This refers to GPs informally supporting each other on an informational (advice) and emotional (venting and reflection) basis.

**Aim:**

To explore GPs’ perceptions of how IPS manifests in the primary care setting and what factors influence effective GP engagement with IPS.

**Design & setting:**

A qualitative study utilising semi-structured interviews to develop an in-depth understanding of GPs’ perceptions of IPS, based on their experiences in practices across England.

**Method:**

Fifteen GPs were purposively sampled to include the views of locum, salaried, and trainee GPs and GP partners. Semi-structured interviews were conducted, recorded, and transcribed verbatim. Transcripts were analysed using inductive thematic analysis.

**Results:**

Four types of IPS were identified relating to emotional support, professional advice, sharing of workload, and mentorship, which reflect existing literature. The frequency and efficacy of IPS was found to be influenced by several factors categorised into individual traits, practice culture, and occupation.

**Conclusion:**

The results highlight where efforts should be directed to improve GP engagement with IPS. Specifically, GP leaders have an important role in shaping practice culture and fostering an environment for IPS to occur. Practices may also benefit from introducing professional development measures targeted at training GPs to better support each other based on their individual traits.

## How this fits in

In relation to the GP workforce crisis, there is growing evidence supporting the effectiveness of peer support in mitigating burnout among healthcare professionals. While research exists on formal peer support interventions, for example, Schwartz Rounds and Balint groups, less is known about informal strategies that have potential to be more effective. This in-depth qualitative interview study explores GP perspectives on the methods and moderating factors of informal peer support (IPS) in general practice. Evaluating the views of GPs will guide future approaches to IPS interventions aimed at managing work-related stress in primary care.

## Introduction

GPs are typically the first point of contact for patients accessing health care in the UK. In recent years, GPs have struggled to cope with the rising demands of their profession with moderate to high levels of GP burnout existing worldwide.^1^ Burnout is the result of excessive work-related stress and has been defined by three subscales: emotional exhaustion, depersonalisation, and lack of personal accomplishment.^2^

A recent report by the General Medical Council (GMC) revealed that more than half of UK GPs (55%) felt they were unable to manage their workload, and 62% found it difficult to provide sufficient care to patients each week.^3^ As a result, GPs are facing higher levels of work-related stress and a reduced sense of personal accomplishment. This is considered a major contributor to the GP workforce crisis illustrated by a reduction in the equivalent of 2078 fewer fully qualified full-time GPs in the UK between September 2015 and January 2023.^4^

Burnout interventions are largely stratified into two types: individual and organisational. There is a consensus that combined interventions are more effective at combatting burnout as they are more likely to be sustainable and exert greater influence.^5,6^ One such approach is to promote supportive interactions between colleagues, termed peer support. Peer support can be defined as the mutual exchange of help between similar others through a range of approaches such as informational (advice) and emotional (venting and reflection).^7,8^

Peer support has been documented in the literature as a means of mitigating burnout and work-related stress in healthcare settings.^9^ It can be grouped into formal methods, which are organisationally mandated and tend to be facilitated by trained peer supporters, or informal methods, referring to spontaneous interactions between colleagues.

Current research on peer support in primary care is based on formal peer support interventions for clinicians, such as Balint groups,^10–12^ Schwartz Rounds,^13^ and one-to-one peer support sessions.^14^ IPS strategies are under-researched since they are difficult to measure,^15^ although are speculated to be more important in burnout mitigation.^16^ Therefore, this qualitative study aimed to investigate the current landscape of IPS and identify the factors that influence engagement with IPS among GPs. In doing so, it highlights modifiable factors that can be acted on to promote engagement with IPS in general practice.

## Method

### Design

This was a qualitative study that utilised semi-structured interviews with GPs as its sole mode of data collection. Methodology was reported in concordance with the Standards for Reporting Qualitative Research.^17^

### Recruitment

Recruitment advertisements were circulated to GPs across England via social media networks (X [formerly known as Twitter] and LinkedIn), personal contacts, and word-of-mouth. Around 30 GPs expressed interest in participation. A purposive sampling strategy was employed to achieve maximal variation in practice location and size, and GP characteristics (sex, GP role, and years of registration). This allowed investigators to collect perspectives from locum, salaried, and trainee GPs as well as GP partners. It was ensured that participants from the same practice were not recruited. Ultimately, 15 interviews were conducted. All selected GPs were provided with a participant information sheet and consent form.

### Data collection

Interviews were conducted between February 2022 and September 2022 over the video-conferencing platforms, Zoom and Microsoft Teams. Each interview was conducted by at least two interviewers to achieve investigator triangulation. Interviewers were medical students who have been trained in interview practice methods. A flexible interview schedule (see Supplementary Box S1) was developed after two pilot interviews and independently reviewed by the study’s supervisor (MF), whose expertise lies in qualitative research methodology. The schedule was designed to be open-ended to allow participants to speak freely, producing richer data.^18^ Interviews averaged 30–60 minutes and were both video- and audio-recorded. Field notes were recorded. The interviews were then transcribed verbatim for analysis, with two other researchers manually checking through transcripts and recordings. Each recording was anonymised, securely stored, then discarded once transcription was complete to ensure that participant confidentiality was maintained.

### Reflexivity statement

Interviews were conducted by three medical students, each with some exposure to general practice via clinical placements. Before starting interviews, investigators explored their preconceived biases on team dynamics in general practice. Consequently, the interview schedule was amended to eliminate leading questions. Interviewers asked open-ended questions and responded to interviewees’ cues allowing for free expression. Thus, improved understanding of participants’ nuanced perspectives also mitigated the potential power imbalance between the interviewers and participants.

### Data analysis

Thematic analysis was executed using the Gioia methodology.^19^ Three researchers (MS, HD, and AH) generated codes using Taguette qualitative analysis software, which were transformed into first order concepts. Using the principles of axial coding, language that was closely associated with the interview transcripts was used to name concepts. These concepts were used to build second order themes, which were then grouped according to the overarching level they act. Themes were reviewed at a team level. Adjustments were made to ensure that data maintained internal and external homogeneity.^20^ Finally, ‘punchy’ names that captured the essence of each theme were curated.^21^ After 15 interviews, the investigators noticed significant repetition of data with minimal development of new theoretical insights. Hence, it was deemed that theoretical saturation had been reached,^18^ and conducting further interviews would be of limited use.

## Results

Fifteen GPs were interviewed across England (Table 1), the majority of whom were male (53%) and GP partners (47%). Participants had been registered practitioners between 2 and 38 years (mean 14.8 years [standard deviation {SD} 11.0 years]) and had been working at their current practice between 3 months and 20 years (mean 6.85 years [SD 5.94 years]). Interviewed GPs were from seven of nine geographic regions in England, with most (67%) working at practices with ≥5 GPs. A total of 13 out of 15 GPs identified IPS as a means of managing burnout in their profession without prompt.

**Table 1.** Participant demographic characteristics (*N* = 15)

GP characteristic		*n*
Sex	Male	8
	Female	7
Years of registration	1–5	5
	6–10	2
	>10	8
GP role	Partner	7
	Salaried	3
	Locum	3
	Trainee	2
Practice location	London	6
	North East England	1
	North West England	1
	Yorkshire and the Humber	1
	East Midlands	1
	West Midlands	1
	East of England	4
Number of GPs at current practice	<5	5
	≥5	10
Years working at current practice	1–5	9
	6–10	2
	>10	4

Emerging themes were grouped under the following two separate research questions:

1. What types of IPS do GPs find effective?
2. What factors moderate effective GP engagement with IPS?

The primary research question identified the following four themes representing the types of IPS between GPs: emotional support; professional advice; sharing workload; and mentorship (Table 2).

**Table 2.** Types of informal peer support that exist between GPs

Type of informal peer support	Definition	Example	Participant expressing theme, *n*
Emotional support	Sharing emotional burden with colleagues, primarily in relation to challenging work-related experiences	*‘Just ranting to them for even five minutes helps.’* (GP12)	13
Professional advice	Informal guidance and reassurance from peers about work-related matters	‘*If there’s something we’re not too sure about, we can run it past each other.’* (GP13)	13
Sharing workload	Redistribution of work-related tasks among colleagues	*‘If one person is getting very stressed and feels overwhelmed, we pool the work.’* (GP2)	11
Mentorship	Younger, less-experienced GPs gaining advice and support from more knowledgeable, senior GPs	*‘What would help is having a mentor, a colleague, where some time could be blocked off to speak to them.’* (GP14)	7

The identified methods of IPS have been previously documented among physicians. Studies have described doctors seeking support from their peers on an emotional and professional basis, such as when facing litigation or managing adverse events in patients.^22,23^ IPS has also been mentioned as taking the form of mentorship.^14^ Sharing workload is commonplace among GPs particularly.^24^

The novel aspect of our findings refers to the identification of factors that influence the predefined types of IPS. These will be termed moderators of IPS. These moderator themes have been categorised by their level of action: individual; practice; and occupation. They have been further divided into sub-themes (Table 3).

**Table 3.** Moderators of informal peer support in GPs

Themes	Sub-themes
Individual	Approachability
	Self-perception
	Relatability
	Familiarity
Practice	Culture of camaraderie
	Shared space
	Psychological safety
	Protected time
	Leadership
Occupation	Job demands
	Siloes
	Hierarchy

### Individual

Individual traits refer to characteristics that are intrinsic to GPs, influencing how well they can support their peers and receive support themselves.

#### Approachability

GPs were more likely to receive support from colleagues they deemed *‘approachable’* (GP1, GP4, GP5, and GP8). These individuals were described as open, easy to talk to, and empathetic, seeming genuinely concerned about their colleague’s wellbeing:

*‘They're the sort of colleague who you would have no hesitation knocking on their door, and they'll always have time to chat. Always very friendly, always happy to listen.’* (GP7, male, salaried GP)

They were also active listeners, reflected in their detailed and honest advice. GPs appreciated when their peers were perceptive and non-judgmental, valuing them as humans with lives outside the practice. They favoured seeking support from peers who were perceived to handle their own stresses effectively and did not appear overburdened:

*‘The colleague who is more level-headed and doesn’t get stressed so easily would be a better person to ask for help.’* (GP1, female, GP partner)

#### Self-perception

Some participants recognised their own fallibility, and this facilitated support-seeking behaviour:

*‘I think I've learnt over time that the most dangerous thing you can do as a doctor is be too proud to ask for help. For people who make mistakes, often that’s the reason why; it’s that they were kind of a bit embarrassed. They didn't ask and then things went wrong. So, I think I've always been quite happy to admit I don't know something.’* (GP12, female, GP trainee)

Equally, they realised the limitations of the support they were able to provide to their peers despite their desire to:

*‘The ideal is to go and help them* [GP colleague] [...] *but obviously you need to have that emotional reserve yourself.’* (GP11, female, locum GP)

In contrast, those who perceived themselves to be invulnerable believed that they could overcome all of their problems on their own, without seeking help from others. These GPs were identified as experiencing more difficulty accessing support from peers as they were less likely to ask for it, and less receptive of support. Instead of viewing support from colleagues as adjunctive, it was perceived as criticism of one’s capabilities:

*‘The thing is, I have a confidence that I'm right. And obviously I'm not always right. I'm mature enough to realise that but there’s a part of me that if someone questions my judgement, I don't like it.'* (GP9, female, salaried GP)

#### Relatability

GPs mentioned finding it easier to be supported by individuals they could relate to through occupying a similar role or having shared experiences:

*‘The three of us or three partners are all women and we're all mums … So, we do talk about our children a lot and the problems we've had with them, partly on a chatty basis. But then sort of also on a practical and help basis.’* (GP4, female, GP partner)

A lack of relatability between peers was demonstrated as a barrier to seeking support, especially if there was a professional boundary between GPs:

*‘I do find it more difficult to ask for help from people that we also employ. Because the salaried doctors tend to be an awful lot younger than we are and less experienced … I do really try hard to keep that gap between us. So, it’s almost like a one-way thing. So, I think I do feel I'm not too bad at supporting them but equally I don't feel that comfortable with baring my soul and telling them all my problems because I do feel there has to be a bit of a gap between us.’* (GP4, female, GP partner)

Occasionally, senior GPs were desensitised to stressful situations affecting their junior counterparts and thus neglected to provide support when indicated:

*‘I think sometimes as you get further on, you forget how much that stuff* [adverse patient event] *affects people. Not that it doesn't affect me, but not to the same degree as it used to. So, you forget that people do need support in those situations.’* (GP3, male, GP trainee)

#### Familiarity

Fostering personal relationships with GP colleagues over a significant period of time made GPs feel better equipped at responding to each other’s nuanced problems. This facilitated their ability to exchange support:

*‘The people who are able to help me out at work, generally, I've known for a very long time. So, we know each other well, we know what the other person would need, emotionally and practically.’* (GP5, male, GP partner)

However, a few participants outlined that familiarity can also act as a barrier to receiving support from certain colleagues due to fear of overburdening them and damaging relationships:

*‘When you've got a problem, you don't want to burden all your friends with it … what you need is someone else, not your partners, but someone from another practice. You don't want to let down your friends and colleagues. And in a GP surgery you've been working at 20 years, then yeah, you’re friends with a lot of people there.’* (GP9, female, salaried GP)

### Practice

Practice-specific factors include both physical and intangible aspects of the practice that shape its environment.

#### Culture of camaraderie

Some practices had a ‘culture of camaraderie’ describing a sense of mutual trust and friendship among colleagues. GPs within these practices felt that they could rely on their peers and comfortably perform spontaneous acts of kindness towards them, knowing that they would be reciprocated. Such a culture was found to be a facilitator of IPS:

*‘I'd offer to bring them* [GP colleagues] *a cup of coffee … So those sorts of things, small acts of kindness, and support are very good at cementing relationships and creating a nice sense of camaraderie and solidarity … because if you’ve received kindness, you’re much more disposed to be kind to others.’* (GP10, female, GP partner)

#### Shared space

Many participants perceived a shared space as a place to *‘escape’* (GP6) from their working environment as well as facilitate better IPS. A dedicated area at the practice made it easier for informal interactions with colleagues to take place, which contributed to how supported GPs felt by one another. It was important for this shared space to be easily accessible to GPs as participants stressed the impact this had on the effectiveness of the space:

*'A shared place to have a break together is a big win. I know several people have chosen a practice that offers that, over a practice that pays more but doesn't offer that, because they find it so valuable.'* (GP3, male, GP trainee)

#### Psychological safety

Psychological safety is the ability to express oneself without fear of humiliation or negative consequences.^25^ A lack of psychological safety and trust between GPs at a practice was discovered as a barrier to engaging with IPS. This effect was heightened in certain groups of GPs, especially less qualified GPs, who had concerns about damaging their reputation so would avoid asking for help:

*‘Some younger GPs, I can see this in my own practice, feel uncomfortable seeking help, reassurance or support from colleagues because they are worried about losing face by doing so.’* (GP2, male, GP partner)

The same was found to be true for locum GPs:

*‘I think as a locum I probably wouldn't be as forthcoming with disclosing my embarrassing moments other than having dealt with it.’* (GP8, male, locum GP)

#### Protected time

Protected time refers to time allocated during the working day, specifically for the purpose of colleagues supporting one other. This was identified as a facilitator of IPS:

*‘We basically had a coffee break every morning which is pretty much compulsory, you have to go for coffee, you can't say you're too busy, although you know people do but actually, our kind of culture is everyone has to have a coffee break at the same time together to support each other and that’s basically how I was supported.’* (GP4, female, GP partner)

*‘I think having allocated time to do that* [support colleagues]*. So, my colleague who I mentor, I have blocked off time for that, so I can do that.’* (GP7, male, salaried GP)

Without protected time, some GPs considered the opportunity cost of engaging with IPS to be too high.

#### Leadership

Some participants demonstrated the significance of strong leadership in creating an organisational climate for IPS to take place. One participant outlined how leaders behave as role models and are responsible for promoting a supportive environment:

*‘If you instil a culture where you're supporting other people, you'd like to think they may do the same, and it will spread around. But it does take somebody to actually start kind of behaving a certain way to enact that culture.’* (GP7, male, salaried GP)

### Occupation

Occupational factors are intrinsic to the role of a GP and tend to exist across practices.

#### Job demands

The lack of time and high workload in primary care was identified as a major barrier to IPS by all participants. Many participants expressed a desire to further support their fellow GPs but explained how it was incompatible with their job demands:

*‘You rarely find yourself with spare time to think for even yourself, never mind thinking about other people.’* (GP3, male, GP trainee)

Owing to this imbalance, GPs prioritised self-preservation and even exhibited a *‘selfish’* (GP3) attitude at times:

*‘There are times where I have felt a little bit stressed, overworked* […] *you just want to look after yourself and just deal with your own stuff. So, you are less likely to go step out and proactively help out.’* (GP7, male, salaried GP)

#### Siloes

By nature, GPs are physically confined to their individual consultation rooms for a large portion of the day. This acts as a structural barrier to GPs supporting each other, reflecting a siloed working environment. Combined with a high workload, GPs are disincentivised to leave their consultations rooms, even when the opportunity arises:

*‘It feels more of an ask for someone to come leave that room, come to your room, or vice versa. They might have a patient in, or you might have a patient, and it’s much more of a dance for that to align correctly. You can't just wander across as easily, so I think that’s a bit of a barrier.’* (GP3, male, GP trainee)

#### Hierarchy

There is a hierarchy that exists in primary care that not only factors in experience levels, but also the complex dynamic between GP partners, salaried GPs, and locums. In some respects, this hierarchical structure was found to motivate more senior GPs to mentor their trainees. On the other hand, this system impedes IPS between GPs of different ranks owing to the aforementioned impact on relatability and psychological safety:

*'A true peer in the sense that she's* [colleague] *also a salaried GP like myself, as opposed to a partner. I think, where I work, there's a bit of a gap, a difference, it's like an us-and-them mentality, rather than someone who is completely equal.'* (GP7, male, salaried GP)

## Discussion

### Summary

**Figure 1.**
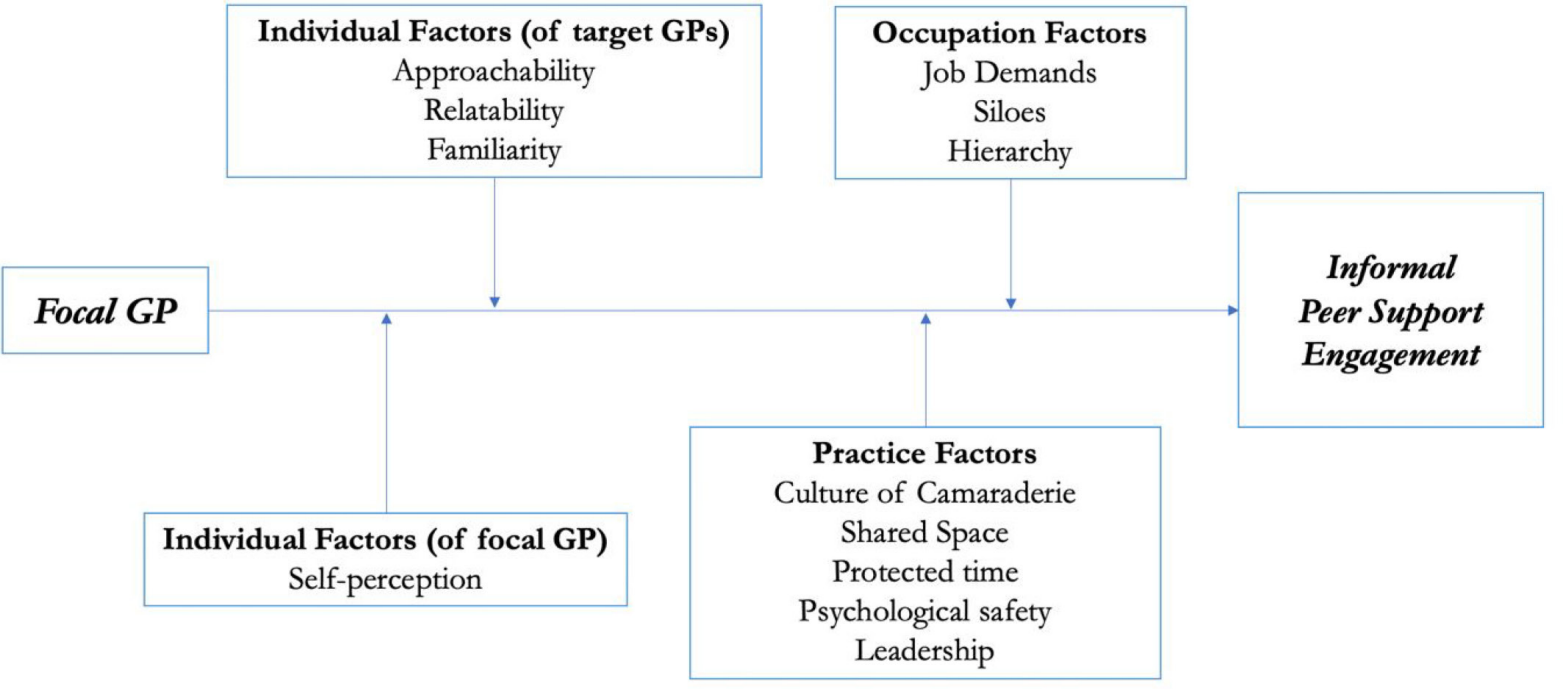
Conceptual diagram of the moderating factors of GP engagement with informal peer support. Focal GP = the individual seeking support. Target GPs = individuals sought to provide support.

This study set out to investigate GPs’ perspectives on the methods and moderators of IPS in general practice and found that participants presently engage with this approach to manage work-related stress. Reflecting on what moderates this engagement, the individual traits of participants determined whether, and from whom, IPS was sought. However, the frequency of seeking or providing support to others was highly impacted by the practice culture and occupation itself. Notably, an open, psychologically safe practice culture was seen as vital in promoting supportive interactions between colleagues. GPs noted that the increasing job demands and intrinsically siloed and hierarchical nature of their profession were inhibitory to the provision of IPS. A diagrammatic summary of the moderators of GP engagement with IPS is illustrated by Figure 1.

### Strengths and limitations

Many clinicians who initially responded to outreach were ultimately unable to be interviewed owing to their significant workloads. Therefore, the study may have attracted individuals who were better supported at work, potentially leading to overemphasis of certain themes. Investigators determined that theoretical saturation had been achieved after thematic analysis of 15 interviews. It is important to recognise that this is subjective, and given the small sample size, conducting further interviews would have fortified the study’s conclusions.

The use of qualitative interviews allowed for extensive discussion and understanding of GPs’ individual experiences, allowing the nuances of IPS to be captured. As the interviewers were medical students unaffiliated with the practices, a further level of anonymity was added when participants were speaking about practice-specific incidents. This meant that the data collected were candid with a wider range of themes elicited. Interviews were conducted on Zoom and Microsoft Teams for convenience. However, this may have invited a greater risk of misinterpretation owing to the loss of non-verbal cues.^26^ To mitigate this, each interview was conducted by at least two interviewers to achieve investigator triangulation, known to improve overall credibility.^27^

The selected sample was diverse in nature, varying in sex, GP role, years of registration, and practice location. Their practice populations, when stratified according to local authority district, had varying Index of Multiple Deprivation rank of average scores, reflecting significant variation in the socioeconomic status of the practice population (see Supplementary Table S1). The mean ratio of GPs per 10 000 population in our sampled GPs’ practices was close in number to the mean ratio for all English authorities (see Supplementary Table S2). This means that the insights derived from the study could be seen as transferable to the wider population in England.

It should be acknowledged that 40% of GPs interviewed were based in London boroughs, which were less deprived on average compared with other regions in England (see Supplementary Table S1). This could mean the study understates the views of GPs working in areas of high socioeconomic deprivation who face specific challenges that increase their risk of professional burnout and ability to engage with IPS.^28^ Arguably this is offset by the fact that the average ratio of GPs per 10 000 population is lower in London (see Supplementary Table S2), which in turn contributes to increased workload and work-related stress.

Despite efforts to ensure a varied sample, most participants belonged to practices with ≥5 GPs or had been working at their current practice for ≤5 years. Nevertheless, 10 out of 12 moderators of IPS were mentioned by GPs irrespective of their practice size and how long they had been working there. These moderators were considered to be generalisable to other GP practices. Siloes was the only moderator not mentioned by GPs working at practices comprising <5 GPs, suggesting that lone office working may not be a barrier to IPS in smaller practices. Similarly, leadership was not highlighted as a moderator by GPs who had been at their current practice for >5 years. This suggests that GPs working at a practice for an extended period may not attribute leadership as a significant factor for IPS owing to a longstanding familiarity with the organisational culture. They might prioritise other aspects of workplace dynamics or personal relationships with colleagues over the explicit influence of leadership. Moreover, a sense of routine or complacency could diminish their recognition of how leadership contributes to fostering a peer-supportive environment.

### Comparison with existing literature

The Health and Social Care Committee report, *Workforce Burnout and Resilience in the NHS and Social Care,* emphasised the importance of *‘compassionate leadership’*, defined by The King’s Fund as *‘leaders listening with fascination to those they lead* […] *and then taking action to help or support them’* in cultivating a supportive workplace culture.^29^ The current study concurs with this but adds that strong leadership also provides their workforce with impetus to participate in IPS behaviours.

Cultivating familiar and relatable relationships were important moderators for engagement with IPS. Literature supports this; for example, near-peers have been documented as the most popular source of social support, surpassing support offered by institutional schemes and mental health professionals.^22^ This study contributes to existing literature by uniquely identifying that a subset of participants would prefer to have relatable colleagues, but who are not necessarily familiar to them, owing to fear of overburdening. This suggests that IPS networks for GPs may benefit from diversity, with members from different practice locations.

Job demands were acknowledged as a barrier to providing IPS. The choice between leaving work on time or supporting their peers was seen almost as an ultimatum, with the latter representing too high of an opportunity cost. This is well documented, with scarcity of time identified as a barrier to peer support in many studies,^30,31^ and a determinant of poor attendance to peer-support sessions.^32^

The following four novel factors of effective IPS were identified: self-perception; culture of camaraderie; siloes; and hierarchy. This study corroborates others revealing that doctors perceive themselves to have a high level of personal resilience,^33^ stressing *'the need for perfection and a deep perception of personal invulnerability*'.^34^ This personality trait has been described as a contributing factor for diagnostic error in medicine.^35^ However, the present study stipulates that perceived invulnerability can manifest as GPs having a higher threshold for seeking support from their peers, paradoxically resulting in a state of burnout. There is an indication to shift the focus from GPs having individual resilience to recognising and extracting value from interdependence.

Additionally, this study introduces the notion of a culture of camaraderie that facilitates IPS. This is supported by Hu *et al* ^22^ who found that establishing a culture of trust is paramount in physicians engaging with peer support to overcome emotional stressors. Interview results showed that colleagues, typically new recruits and locum doctors, who failed to conform with the established culture of camaraderie were removed from benefits associated with reciprocal relationships. Hence, caution should be taken to promote a culture of IPS that is inclusive of all types of GPs.

Moreover, the siloed nature of primary care poses a physical barrier for circumstantial interactions between colleagues that are commonplace in hospital environments. This suggests greater effort is required to cultivate an environment for these interactions to occur; for example, via protected space and time.

GP settings inherently have a hierarchical organisational structure, with GP partners occupying the most senior positions. Rigid hierarchy has been documented as a barrier to engagement with IPS. A Dutch study identified that medical residents were significantly more dissatisfied with the social support provided to them by their supervisors compared with their near-peers, which the present findings support.^36^ However, the literature fails to shed light on social support strategies for senior clinicians. This study identifies that senior GPs also face similar challenges to their junior counterparts. They were reluctant to seek support from their juniors, perceiving it to undermine their role as leaders and supervisors. Consequently, GP partners may find it difficult to access IPS in their practices and may require specifically designed interventions.

### Implications for research and practice

To the authors’ knowledge, this is the first study to explore IPS and its moderating factors in general practice in England. This study is of relevance to GPs, policymakers, and partners as it provides basis for evidence-based interventions to facilitate better IPS in primary care, thus minimising GP burnout in a cost-effective manner. GP leaders, in particular, have a responsibility to implement strategies that encourage a conducive environment for IPS, such as advocating for protected time and shared spaces. It should be noted that this may constitute an additional burden to a subset of GPs who already suffer high levels of work-related stress.^37^ Future research should quantitatively assess the effects of improving the practice environment on engagement with IPS to lend further credibility to these qualitative findings.

All participants in this study affirmed that they had no training on peer support concepts in their careers, instead learning informally through observation. Therefore, there may be a niche for the development of a peer communication curriculum that aims to cultivate the individual traits of GPs, making them better equipped to engage in IPS. One study’s peer communication curriculum received positive appraisals by clinicians on their peer-to-peer role-playing scenarios (where they employed a ‘distressed’ and ‘helper’ clinician).^38^ This represents a low-cost intervention that could be introduced as a necessary part of professional development.

With growing patient needs and a declining GP workforce,^39^ GPs are facing intense workloads paired with substantial time constraints. This is confounded by recent healthcare reform that has placed an increased onus on primary care to manage long-term conditions in the future.^40^ It is important to note that improving IPS alone will not eliminate burnout; however, it is an important step in optimising team dynamics in general practice, paving the way for a more cohesive and sustainable workforce.
